# Complaints filed with the Veterinary Disciplinary Board in Sweden – a thematic analysis

**DOI:** 10.1186/s12917-026-05540-6

**Published:** 2026-05-09

**Authors:** Ida Svanberg, Agneta Egenvall, Karolina Brunius Enlund

**Affiliations:** Department of Clinical Sciences, Swedish University of Agricultural Sciences, Box 7054, Uppsala, 750 07 Sweden

**Keywords:** Veterinary communication, Qualitative analysis, Professional misconduct, Client perceptions

## Abstract

**Background:**

This study examined complaints submitted to the Veterinary Disciplinary Board for Animal Health Staff in Sweden to identify reporting reasons, case outcomes, and recurring themes. A quantitative compilation and qualitative thematic analysis were conducted on 500 disciplinary cases processed 2021, 2022 and 2023 (*n* = 86, *n* = 227, *n* = 187).

**Results:**

Of 500 cases, 87% did not lead to sanctions, of which 21% (*n* = 106) were fully dismissed. The sanctions were admonitions (*n* = 48; less severe), warnings (*n* = 7; more severe), probations (*n* = 3) and revoked licence (*n* = 1). Veterinarians accounted for the majority of reported animal health staff (522/608).

Thematic analysis identified three overarching reasons for reporting: clinical errors and quality of care (such as perceived mistakes in diagnostics, treatment, or examination), professionalism and ethics (including e.g. perceived lack of empathy towards owners or animals), and communication and information (including e.g. perceived loss of autonomy). These themes illustrate that while clinical competence is central, non-medical aspects play a critical role in owners’ perceptions of care. A limitation of thematic analysis is its subjectivity; different researchers may identify different themes in the same data due to interpretative differences.

**Conclusion:**

In conclusion, prominent sub-themes were linked to non-medical issues, underscoring the need for effective communication and professionalism to prevent conflicts and build trust between veterinary staff and animal owners.

## Background

Veterinary disciplinary boards for animal healthcare professionals play an important role in ensuring legal certainty and maintaining public trust in veterinary practice [1–3]. By overseeing professional conduct and evaluating complaints, the boards help guarantee that animal health professionals adhere to current laws and regulations, and that instances of malpractice are identified and addressed. However, particularly unfounded or excessive complaints can place considerable psychological stress on individual veterinarians, potentially leading to anxiety, distress, and negative mental health outcomes, as well as affecting hospital practices [4, 5].

In Sweden, The Veterinary Disciplinary Board for Animal Health Staff (VDB) is a statutory authority established under national legislation governing animal health care professionals. The VDB reviews complaints against animal health staff based on breaches, intentionally or negligently, of legally defined professional duties and may impose disciplinary sanctions for medical errors [1, 6, 7]. The VDB consists of a chairperson, who must be or have been a judge, and seven members, all appointed by the government. At least two of the members must be licensed veterinarians [1], while the others may be selected from individuals with knowledge of animal care who represent the public interest.

The VDB examines cases exclusively against animal health staff, which includes primarily veterinarians and licensed veterinary nurses, but also certified farriers and veterinary approved licensed physiotherapists, dentists and nurses [1]. Only supervisory authorities and animal owners or keepers are authorised to report members of the animal health staff to the VDB. The VDB assesses the nature and seriousness of the alleged error and may issue formal disciplinary measures in increasing severity: an admonition (“erinran”, less severe), a warning (“varning”, more severe), a probationary period, or revocation of the license to practice [1].

The entire reporting process is conducted in writing. When a report is received, it is first checked to ensure it meets formal requirements, including a two-year statute of limitations from the incident until the reported person is informed. If the report is incomplete or does not regard a veterinary medical error, it is dismissed without review [1].

If accepted, the report is sent to the reported individual, who must respond in writing within a set time [1]. The reported person’s response is then forwarded to the complainant, starting a written exchange where all documents are shared. The documentation is then sent to the Swedish Board of Agriculture (Jordbruksverket), the central government authority which provides input and guidance based on regulations and oversight responsibilities. After that, both parties may submit final comments before the VDB makes an independent decision. The decision becomes official when sent in writing to both parties [1]. The process to reach a decision takes about one year. If either party (the reported person or the complainant), or the Board of Agriculture, believes the decision is incorrect, it can be appealed to the Administrative Court in Jönköping [1].

Veterinary disciplinary systems in other countries have both similarities and differences compared to the Swedish system. For example, in both Sweden and the Netherlands, the VDB is a governmental body [8] as opposed to in the UK, where the VDB is a statutory professional body and operates independently from direct government control. In the UK and some states in the US, complaints are first evaluated by a committee before being taken into consideration [9–11], in contrast to the Netherlands where almost all complaints are taken into consideration [8]. In addition, procedures of the state veterinary medical boards in the US may differ between states [10]. In Denmark and Norway, the VDBs’ decisions are not legally binding and serve only an advisory function, unlike in Sweden [12, 13].

The aim of this study was to analyse complaints submitted to the VDB by summarizing quantitative data, identifying reasons for reporting, dismissals, and sanctions, and examining these reasons through thematic analysis. Both the complaints leading to sanctions and those that did not were included in the study.

## Methods

The study analysed disciplinary cases received by Sweden’s VDB during 2021–2023, including dismissed cases. The documentation from the 500 most recently closed disciplinary cases, which are classified as public records under Swedish freedom of information law, was requested on August 27, 2024, and received on August 29, 2024. The data can be publicly accessed by requesting the complaint records from the VDB [1]. As these records constitute public data, no ethical review is required under Swedish legislation. Personal data were documented during the analysis solely to address specific research questions, such as whether the same person had been reported multiple times. These 500 cases included 187 cases from 2023, 227 cases from 2022 to 86 cases from 2021.

A combination of quantitative descriptive compilation and qualitative thematic analysis was conducted based on the information in the documentation of the disciplinary cases.

### Quantitative data

Descriptive data, including the reported individual’s name, gender, profession, the complainant’s identity (including whether private individual or supervisory authority), the VDB’s decision and primary justification, whether the reported individual disputed or admitted the allegations, and the use of external experts, were documented in Microsoft Excel. Interpretation was minimal, with data compiled as stated in the source material.

### Thematic analysis

The analysis was conducted using a standard thematic analysis approach [14]. An initial superficial analysis of ~ 30 cases was conducted to familiarise with the material and refine the analytical approach. These cases were later reanalysed in-depth when all cases were analysed. Cases resulting in sanctions (deemed by the VDB as involving medical errors), non-sanctioned cases, and dismissed/closed cases were separated in the analysis. Cases were summarised, coded, and analysed for key phrases (e.g., reasons for dissatisfaction, medical errors, relevant outcomes, contextual factors). Codes were grouped into sub-themes and merged into overarching themes by authors IS and KBE through frequent discussion to ensure the best understanding of the data and emerging findings. The analysis was latent and inductive, allowing interpretation of implicit information without predetermined hypotheses [14].

The written nature of complaints eliminated the need for transcription, though case summaries used in the study were provided by the VDB and likely involved some degree of interpretation from their side. The study did not use any potential medical records, emails, or receipts referenced in cases.

Most disciplinary cases involved more than one sub-theme and/or theme. Similarly, multiple individuals can be involved in a single case, and the same individual may appear in multiple cases. Thus, the total counts of sub-themes/themes and individuals do not match the total number of cases analysed.

## Results

A majority of the 500 cases, 436 (87%), received no disciplinary action, of which 106 cases were fully dismissed. Of the 13% (*n* = 64) that received sanctions, 48 were admonitions, 7 warnings, 3 probations and 1 had a revoked licence.

### Background data

#### Reported individuals

In the 500 cases, a total of 603 individuals were reported. Of these, 446 were women, 154 were men, and in three cases gender information was not provided. Sixty-nine individuals were reported multiple times. Of these, 52 individuals had two complaints filed against them, 15 individuals had three complaints, and two individuals had six complaints. The remaining 534 individuals were each reported once.

Most commonly, the complaint was directed at a single person (*n* = 369), followed by two individuals (*n* = 65). Cases involving eight or more individuals occurred three times, with the highest number being 14 people reported in a single case.

The reported individuals contested the complaint in 367 of 394 adjudicated cases (93%). In 11 cases, the allegations were fully admitted, and in five cases, they were partially admitted. In six cases, no response was received in full or in part.

Clinically active veterinarians were the most frequently reported group, with 522 practising veterinarians, including 27 veterinarians temporarily licensed prior to full certification. Licensed veterinary nurses followed with 33 cases, and six certified farriers were also reported. One individual belonged to two of these professions.

#### Complainants

Most cases involved a conflict between a private individual and one or more animal healthcare professionals. Thirteen private individuals filed multiple complaints—10 twice, and 3 three times. In 18 cases, the complainant was a supervisory authority: the Swedish Board of Agriculture (seven cases) or a County Administrative Board (11 cases). The County Administrative Board (Länsstyrelsen) investigates animal-welfare complaints and carries out inspections, and may report animal health staff suspected of professional misconduct or negligence. Five cases lacked a listed sender and were therefore dismissed.

Three cases involved individuals requesting revocation of their own certification (certified farrier, *n* = 1) or prescription rights (veterinarians, *n* = 2). Another case concerned an application to reinstate a previously revoked license. All these requests were approved and are not included in the analysis.

#### Reason for visit

The most common visit type among the 500 cases was a scheduled appointment (278 cases), including 42 for preventive care (e.g., vaccinations, examinations, nail clipping). Surgical procedures were involved in 42 visits, 27 of which were dental. Euthanasia was listed as the reason for visit in eight cases, but occurred in 83 additional cases as well. Other visits involved varied symptoms such as increased urination/thirst, weight loss, tumours, and itching. Emergency visits accounted for 95 cases, involving for example dystocia, sudden pain, poisoning, or breathing difficulties.

The reason for the visit was often missing in dismissed/closed cases and in complaints from supervisory authorities, where the allegations typically concerned broader or ongoing issues.

In cases where the animal species was specified, 280 involved dogs, 106 cats, 62 horses, nine rabbits or guinea pigs, and five other animals.

### Dismissed cases

Cases can be dismissed from review if the requirements are not met [1]. Cases may also be closed if the complainant chooses to withdraw the complaint; this occurred in 19 cases. A total of 170 cases were either partially or entirely dismissed. Of these, 64 (13% of the total 500) were partially dismissed and 106 (21% of the total 500) were completely dismissed from review by the VDB. In 24 of the 64 partially dismissed cases, a separate, more concise protocol was written in addition to the review summary, outlining, among other things, what was dismissed and why. In the remaining 40 cases, it was instead noted directly in the review document which parts of the complaint were dismissed and the reasons, after which the rest of the case proceeded to review. All fully dismissed cases consisted only of a single protocol.

#### Thematic analysis

Below is the result of the thematic analysis of the reasons for dismissals (Table 1).

**Table 1:** Identified themes and sub-themes among dismissed cases, with clarifications

Theme	Sub-theme	Clarification
Incorrect reason for report	Not animal health staff	The reported person is, for example, an animal caretaker, uncertified farrier, receptionist, animal hospital manager, or official county veterinarian
	Wrong person reported	The reported person was not involved in the case at the time in question
	Not a veterinary medical procedure	The reported person has not performed any veterinary medical procedure in the case, or the report does not concern a veterinary medical procedure, e.g., conduct, pricing, or administrative tasks not covered by legislation (6)
Technical errors	Incomplete report	The report lacks, for example, sender, names of reported persons, medical record, description of what the reporter wishes to have reviewed, or similar information
	Reporter lacks authority to report	The reporter is neither the owner of the animal nor had the animal in their care (caretaker, stable staff, etc.) at the time of the report
	The case is time-barred	Two years or more have passed since the incident, or the reported person cannot be informed of the case in writing before the limitation period expires

### Non-sanctioned cases

A majority of the adjudicated cases, 330 out of 394 (84%), received no disciplinary action.

#### Thematic analysis

Of the cases without disciplinary action reviewed by the VDB, eleven sub-themes and three overarching themes for the reasons behind the complaints were identified (Table 2).

**Table 2:** Identified themes and sub-themes among reviewed cases without disciplinary action, with examples

Theme	Sub-theme	Example
Clinical Errors and Quality of Care	Diagnostic errors according to the complainant	Incorrect, delayed, missed, absent, or contradictory diagnosis
	Incorrect/inadequate treatment, care, and/or intervention according to the complainant	Mistreatment, omitted/unnecessary treatment, delayed treatment, medical errors/complications, poor hygiene, injuries related to care, or aftercare issues
	Inadequate examination according to the complainant	Too brief/quick or absent examination, failure to examine relevant body part/system or whole animal, poor follow-up
	Complications from anaesthesia or surgery	Bleeding, post-operative infection, adverse drug reaction, injuries from intubation, ruptured suture
	Deficiencies in documentation according to the complainant	False certificates, inaccuracies in records, incomplete records
Professionalism, Ethics, and Conduct	Distrust of animal health staff’s knowledge, competence, experience, and/or integrity	Staff seem inexperienced, lack of specialist skills, staff failed procedures, perceived bias, suspected financial motives
	Lack of empathy toward the animal according to the complainant	Insensitive or rough handling during exams, sampling, euthanasia, or care; inadequate pain relief
	Lack of empathy toward the owner according to the complainant	Staff perceived as rude, don’t listen, unprofessional, offensive, deceptive, indifferent to owner’s feelings or perspective
	Costs	Dissatisfaction with costs, request for compensation due to poor handling, treatment failure, or complications
Communication and Information	Perceived loss of autonomy	Staff act against owner’s wishes, owner felt attacked, pressured, threatened, staff performs actions without asking; staff reported owner to the County Administrative Board
	Inadequate/incorrect/misleading information, communication, or recommendation according to the complainant	Regarding prognosis, euthanasia, discharge, complications, or unexpected death; staff don’t speak sufficient Swedish

Among non-dismissed cases where no sanction was issued, the most frequently identified sub-theme was “Incorrect/inadequate treatment, care, and/or intervention according to the complainant”, which refers to perceived malpractice where the VDB either disagreed with the complainant or could not confirm that malpractice had occurred. This was followed by “Inadequate/incorrect/misleading information, communication, or recommendation according to the complainant” and “Lack of empathy toward the owner according to the complainant”.

In a substantial proportion of cases without sanction, multiple sub-themes were identified. In other cases, a single sub-theme was present, most commonly “Complications from anaesthesia or surgery” or “Deficiencies in documentation according to the complainant.” Each sub-theme was, in some instances, identified as the sole reason for a complaint.

#### Criticism without disciplinary action

In 66 cases without sanctions, the VDB and/or the Swedish Board of Agriculture still found the handling of the matter to be suboptimal.

In some cases, minor flaws—such as suboptimal investigation or poor communication—did not warrant sanctions but were noted in comments. No sanction was issued when errors were deemed excusable due to mitigating factors like atypical disease presentation, lack of experience, or circumstances beyond the individual’s control.

### Sanctioned cases

Of the 500 cases, 64 were deemed to involve sufficiently serious shortcomings to warrant a sanction. This corresponds to 13% of the total cases (*n* = 500) and 16% of the fully adjudicated cases (*n* = 394).

Admonitions (the least severe sanction) were issued to 48 individuals (Table 3) across 52 cases. In one case, both reported individuals received an admonition, while in the remaining cases, one person per case received an admonition. Four individuals, all veterinarians, received two admonitions each during the study period.

**Table 3:** Distribution of professions and gender by type of sanction

Sanction	Profession	Gender
Admonition	46 veterinarians,	23 women, 25 men
	1 licensed veterinary nurse,	
	1 certified farrier	
Warning	6 veterinarians,	1 woman, 6 men
	1 licensed veterinary nurse	
Probation	2 veterinarians,	1 woman, 2 men
	1 licensed veterinary nurse	
Revoked license	1 veterinarian	1 woman

Warnings were issued to seven individuals (Table 3) across eight cases; one veterinarian appeared in two separate cases and received warnings in both. One veterinarian received both an admonition and a warning for separate cases, and another received two admonitions and one warning.

Probation was given to three individuals (Table 3), two of whom had been reported previously, and one had received earlier admonitions and warnings. The individual whose license was revoked appeared in only one case.

In the 18 cases submitted by a supervisory authority, a sanction was issued in eight (44%): one admonition, three warnings, all the three probation cases and the license revocation.

#### Reasons for admonitions

When issuing an admonition, the VDB first refers to the legal texts [6], followed by comments specific to the case. Examples of such comments include:

- “The errors are not minor and are not excusable. […] should therefore be given an admonition.”
- “Overall, the board considers that […] has been so negligent in their professional conduct that […] should be given a disciplinary sanction in the form of an admonition.”
- “The errors made are of such a serious nature that […] cannot avoid a disciplinary sanction in the form of an admonition.”

#### Reasons for warnings

Warnings were issued when an admonition was deemed insufficient, such as in cases of repeated similar errors without behavioural change, or deliberate misconduct.

Furthermore, the seriousness of the error is often cited as a reason for the sanction:

- “The mistake and the omission were so severe that […] should be given a warning.”
- Based on the above, the disciplinary board makes the overall assessment that […] has failed in their professional conduct in such a serious way that […] cannot avoid a disciplinary sanction in the form of a warning.”
- “For this very serious negligence and malpractice, […] cannot avoid a disciplinary sanction in the form of a warning.”

#### Reasons for probation and revocation of license

Three cases during the study period resulted in probation (Table 3). Reasons for probation included working beyond formal competence, repeated failure to follow scientific standards despite prior sanctions, intoxication during patient contact, and improper prescribing of narcotics. In one case, probation was issued instead of revocation due to demonstrated insight and corrective action.

The license revocation (Table 3) followed sustained disregard for scientific and hygiene standards, including home surgeries without gloves and improper antibiotic use.

#### Thematic analysis

The VDB’s task is to assess veterinary medical errors. Accordingly, all cases where a sanction was imposed share this common factor. Below are the identified themes and sub-themes for the reasons behind sanctions (Table 4).

**Table 4:** Identified themes and sub-themes among causes for disciplinary action, with examples

Theme	Sub-theme	Example
Failure to practice according to science and proven experience	Incorrect issuance of certificates forming the basis for transactions or government actions	Veterinary inspection/examination prior to sale/purchase or seizure of animals, etc.
	Abuse of prescribing rights	Unfounded prescription of human narcotics to own dog, prescribing antibiotics without culture and sensitivity testing, use of fluoroquinolones without proper diagnostics
	Professional unsuitability	Repeated serious medical errors, clear unwillingness to follow laws and policies, severe lack of knowledge and/or competence, failure to follow scientific and proven practices, abuse of alcohol and/or drugs
Mistreatment or high risk of mistreatment	Insufficient attentiveness or thoroughness	Surgical sponges left in body cavities, use of wrong medication or incorrect dosage
	Inadequate record-keeping with negative consequences for the animal	Complete absence of medical records, missing medication or dosage information, missing or deficient surgical reports, no status recorded before procedures
	Serious mistreatment, misinterpretation of diagnostics, or misjudgement of patient condition negatively impacting the patient	Contraindicated treatment (e.g., steroids in untreated diabetes), erroneous euthanasia recommendation based on false tumour diagnosis, advice that severe pyometra could wait for treatment approx. 1 week
	Working beyond formal qualifications	Licensed veterinary nurse initiates a CT scan without veterinary order or knowledge

## Discussion

This study shows that perceived lack of clinical skills, poor communication, and apparent lack of empathy was the most prominent sub-themes in complaints to the VDB. Unmet client expectations have previously been associated with complaints, particularly regarding professionalism, communication, and fair pricing [15]. Our findings, which include many non-medical sub-themes, align with a study on what pet owners want and expect from their veterinarian, not only clinical competence, but also communication skills, professionalism, and attention to animal welfare [16].

### Reasons for complaints

Complaints primarily related to technical issues often also involved communication or interpersonal problems, consistent with previous research [15, 17–19]. Complaints frequently addressed multiple issues, suggesting that while isolated mistakes may be tolerated, a perception of multiple inadequacies increases the likelihood of filing a complaint. Owners may also include minor concerns to strengthen the perceived seriousness of a complaint.

Among complaints based on a single issue, complications from anaesthesia or surgery were the most common. This may reflect misunderstandings, as complications do not necessarily indicate error [20]. Nevertheless, these findings highlight the need for clearer communication about procedural risks, particularly regarding anaesthesia.

Euthanasia was a common context, appearing in nearly 20% of cases. This aligns with previous studies emphasizing the emotional complexity of euthanasia and the challenges in communicating effectively during such sensitive situations [17, 21].

Perceived clinical errors were a frequent reason for complaints. Both human and veterinary medicine show that many preventable injuries result from systemic factors rather than individual negligence [22, 23]. A “systems approach” focuses on structural weaknesses, such as communication breakdowns or lack of protocols, supporting a culture of learning and safety [24]. Effective communication may also reduce the risk of being reported during adverse events. Previous research indicates that clients value honesty, apologies, accountability, and assurance of preventive measures, even when financial compensation is stated as the desired outcome [25]. Open communication during or after adverse events may thus reduce complaints.

Client complaints can improve patient safety by identifying potential risks, but they can also contribute to so-called ‘defensive practice’ where client demands may come before the animal’s best interests [2, 3]. The key role of the Veterinary Disciplinary Board (VDB) is to ensure professionals adhere to best practices and maintains public trust. Although most complaints do not result in sanctions, the process can be stressful for veterinarians [4, 5, 26, 27]. Transparency, clear procedures, and accessible information help reduce stress and foster trust in the disciplinary system.

### Reasons for sanctions

Only 13% of complaints in Sweden result in sanctions, suggesting that many complaints reflect subjective perceptions rather than verifiable medical errors. In some cases, medical records are incomplete or absent, which in itself constitutes an error. When the complainant’s and veterinarian’s accounts differ, records carry significant weight, making accurate documentation essential. Deficient records can also hinder or prevent assessment, particularly in disputes of word against word. Thus, medical records are crucial both for patient safety and for ensuring legal certainty.

In contrast to our study, studies in other countries report much higher sanction rates—32.8% in New Zealand [15] and 33.3% in Brazil [28]. A study from the Netherlands [29] showed that 37.0% of the client complaints lead to sanctions such as warnings or reprimands from the years 2002–2016. This discrepancy may be due to differences in what types of complaints are examined by disciplinary boards. In addition, some countries have processes that more thoroughly filter complaints prior to review [8–10]. Some differences may also be due to cultural factors such as the status of animals and public attitudes toward them.

The most common reasons for sanctions in Sweden were professional negligence, including poor documentation and certification errors, and mistreatment, consistent with international findings [9, 28]. The presumption of innocence, an internationally recognized principle, likely contributes to the relatively low sanction rate, as cases lacking clear evidence are often resolved in favour of the accused [30]. Consequently, some dismissed complaints may in fact be well-founded but fail due to insufficient evidence, and if this principle is adhered to it may be more likely that an error goes unsanctioned than that a veterinarian is erroneously sanctioned.

### Dismissed cases

Some complaints involved individuals or facilities outside the VDB’s jurisdiction, such as uncertified farriers, animal caretakers, hospital directors, entire veterinary hospitals, zoos, or veterinarians not in clinical practice. These 61 cases highlight the need for better communication regarding the scope of the VDB’s authority.

### Complaints by gender

In Sweden, the gender distribution of reported veterinarians aligns closely with the national workforce, with approximately 75% women [31], in contrast to New Zealand where 70.2% of complaints involved male veterinarians [15], despite women comprising the majority of the veterinary profession at the same time [32]. This suggests that gender does not significantly influence the likelihood of being reported in Sweden.

However, men were overrepresented among those receiving sanctions, 33 out of 64, consistent with previous research on male physicians being overrepresented in disciplinary sanctions [33, 34]. The effect of age may also warrant further investigation, as the median age of male veterinarians is higher than that of female veterinarians in veterinary populations [31].

### Complaints by animal species

Only two cases (0.004%) involved livestock, one cow and one sheep, partly consistent with a U.S. study in which no disciplinary cases involved client complaints about livestock [9]. By contrast, 14.1% of cases in New Zealand involved production animals [15], and in the Netherlands, 13% were related to livestock [29]. The reason for this discrepancy remains unknown.

### Strengths and limitations

This study used thematic analysis [14]. As thematic analysis can be conducted in various ways, leading to different outcomes, this flexibility must be acknowledged. To increase the reliability of the findings, the thematic analysis was performed according to a well-established and widely applied framework [14].

Future research on VDB complaints could further investigate background factors of the complainant, such as how the human-animal bond or personality affects the likelihood of filing a complaint to the VDB. Other relevant factors include age of the complainant [18] and the years of professional experience of the staff member [15], data that were not accessible from the documentation.

## Conclusions

Most reported veterinarians were not found to have committed medical errors. Prominent sub-themes of reasons for complaints were in fact linked to non-medical issues, highlighting the importance of showing empathy and involving owners in decision-making to prevent complaints and build trust between veterinary staff and animal owners. While being reported can be stressful, the complaint process plays an important role in identifying genuine misconduct and safeguarding animal welfare. This study emphasizes that professional competence, respectful behaviour, and effective communication are essential to minimize conflict and stress in veterinary care.

## Data Availability

Data on the analysis available from authors on reasonable request due to privacy/ethical consideration. All VDB complaint cases are classified as public records under Swedish freedom of information law.
